# Mathematical modeling of immune kinetics in advanced cancer through meta-analyses of complete response rates: immune synchronisation emerges as the likely key determinant of clinical response

**DOI:** 10.1186/2051-1426-1-S1-P150

**Published:** 2013-11-07

**Authors:** Martin L Ashdown, Brendon J Coventry, Derek Abbott

**Affiliations:** 1 Medicine, University of Melbourne, Melbourne, VIC, Australia; 2 Surgery, University of Adelaide, Adelaide, SA, Australia

## Introduction

Recent evidence suggests that the failure of the immune system to eradicate cancer cells is due to the tumor co-opting the normal regulatory/suppressive and homeostatic mechanisms of the immune system, thus avoiding immune based destruction. A small percentage of patients with complete responses (CR; no evidence of tumor) result from many systemic therapies. CR’s usually underpin long-term survival.

## Methods

Data from numerous studies using standard chemotherapy, targeted therapies and immune modulatory therapies were mathematically analysed, using meta-analysis (M-A) techniques, to evaluate the Complete Response (CR) Rates.

## Results

68 studies using standard chemotherapy for 13 cancer types treated with 8 drug classes were analysed by meta-analysis and showed a CR rate of 7.4%. A range of targeted therapies (B-raf; Mek) and immune based therapies (CTLA-4; PD-1; PD-L1) were also assessed which showed similar findings. Meta-analysis of 62 IL-2 based therapies showed a similar CR rate also. Over 8000 patients were examined across the meta-analyses.

## Conclusions

Mathematical analyses of over 130 clinical trials, inclusive of over 8000 patients, has demonstrated that the CR rate for patients treated with widely divergent therapies is remarkably fixed at between 5 and 10%. The probability of this occurring by chance alone is extremely close to zero, and is both scientifically and clinically implausible. Therefore, an underlying predictive operative biological mechanism must apply. It is highly likely that the known principles of immune kinetics between effector and regulatory homeostatic functions must therefore determine a degree of immune synchronization to produce these observed CR’s. We have derived a mathematical model and equation using the principles of controlled homeostatic systems, which can be used to explain our meta-analysis findings, and the clinical efficacy.

